# Polypeptide-Nanoparticle Interactions and Corona Formation Investigated by Monte Carlo Simulations

**DOI:** 10.3390/polym8060203

**Published:** 2016-05-25

**Authors:** Fabrice Carnal, Arnaud Clavier, Serge Stoll

**Affiliations:** University of Geneva, F.-A. Forel Institute, Environmental Physical Chemistry, 66 Boulevard Carl-Vogt, 1205 Geneva, Switzerland; arnaud.clavier@unige.ch (A.C.); serge.stoll@unige.ch (S.S.)

**Keywords:** Nanoparticle complexation, polypeptide adsorption, polypeptide corona, acid/base properties, Monte Carlo simulations

## Abstract

Biomacromolecule activity is usually related to its ability to keep a specific structure. However, in solution, many parameters (pH, ionic strength) and external compounds (polyelectrolytes, nanoparticles) can modify biomacromolecule structure as well as acid/base properties, thus resulting in a loss of activity and denaturation. In this paper, the impact of neutral and charged nanoparticles (NPs) is investigated by Monte Carlo simulations on polypeptide (PP) chains with primary structure based on bovine serum albumin. The influence of pH, salt valency, and NP surface charge density is systematically studied. It is found that the PP is extended at extreme pH, when no complex formation is observed, and folded at physiological pH. PP adsorption around oppositely-charged NPs strongly limits chain structural changes and modifies its acid/base properties. At physiological pH, the complex formation occurs only with positively-charged NPs. The presence of salts, in particular those with trivalent cations, introduces additional electrostatic interactions, resulting in a mitigation of the impact of negative NPs. Thus, the corona structure is less dense with locally-desorbed segments. On the contrary, very limited impact of salt cation valency is observed when NPs are positive, due to the absence of competitive effects between multivalent cations and NP.

## 1. Introduction

Serum albumins, the most abundant plasma proteins in the mammalian circulatory system synthesized in the liver, have been a subject of interest for many years. As a result, they are now well characterized and largely involved in fundamental research, biomedical, and industrial applications. Typically, these proteins are involved in binding and transport of a large range of compounds, such as fatty acids, amino acids (AAs), metals, drugs, or inorganic ions [1,2,3,4]. Human and bovine serum albumin (HSA and BSA) display about 76% of sequence homologies with native structures known to be heart-shaped and composed of three homologous domains [5,6]. However, the activity of serum albumin proteins is strongly dependent on target-specific binding and thus on conformational properties. Various physicochemical factors can affect the protein stability, inducing structure changes that lead to denaturation and loss of biological activity.

Depending on solution pH and ionic strength, serum albumin proteins can adopt different conformations, described as extended, fast, native (N), basic, and aged, from acidic to basic pH [7,8,9,10]. The three homologous domains of BSA/HSA have different stabilities in acidic/basic environments and are involved in the protein denaturation process. Moreover, structural transitions have the ability to be reversible with pH variations [11]. The ionic environment effect is an important parameter, since repulsive electrostatic interactions are decreased with salt, thus affecting stability and diffusivity of albumin proteins [12,13]. Conformational changes are also influenced by protein concentration, as reported by Barbosa *et al*. [8] for BSA. At extreme acidic pH, the BSA conformations are found to be unfolded, but a likely molten-globule state, already suggested for HSA [14], is observed with the increase of BSA concentration. Generally, the native structure is not dependent on concentration at neutral pH, but BSA expansion was observed by He *et al*. [15] in ultra-diluted aqueous solutions due to extramolecular hydrogen bonds formed between proteins and water molecules.

The binding process of serum albumins with other ions or molecules affects their structural properties. As protein folding and unfolding is crucial in regulating biological activity [16], abundant research is found in this area using experimental techniques (UV–Vis, circular dichroism (CD), Fourier transform infrared spectroscopy (FTIR), dynamic light scattering (DLS), differential scanning calorimetry (DSC), atomic force microscopy (AFM), small-angle X-ray scattering (SAXS), transmission electron microscopy (TEM), fluorescence spectroscopy, *etc*.). The main driving force involved in BSA adsorption dynamics and conformational changes due to complex formation with charged nanoparticles (NPs) is reported to be long-range (electrostatic) [17]. Thus, the protein surface charge and anisotropy significantly influence the affinity with nanomaterials or surfaces [18,19,20]. However, other short-range interactions, such as van der Waals, hydrophobic, hydrophilic, and structural, also play a role in the formation of complexes [21]. In this context, serum albumin proteins can be found destabilized with silver NPs but stable with gold NPs, which can be used to stop HSA unfolding by ultraviolet radiation [22,23]. Polyampholyte-coated magnetite NPs have also shown a promising behavior in magnetic resonance angiography (MRA) due to the elimination of strong interactions with BSA [24]. Moreover, the NP surface curvature and coverage may modulate the amount of interacting serum albumin proteins, and thus their affinities [25,26]. In this case, BSA proteins show a more pronounced tertiary denatured state at low surface coverage. Carbon nanotubes, which are widely involved in nanobiotechnology and nanomedicine, affect BSA conformational stability, hence modifying the ability to bind ligands and the rate of denaturation or fibrillation [27]. In solution, salt screening effects modify long-range interactions between proteins and NPs, but also impact protein–protein repulsions, which can result in a more efficient fibrillation process of serum albumins [28,29].

Computer simulations, complementary to experiments, represent powerful tools to study in detail specific behaviors of protein conformation or adsorption [30]. As suggested by Shen *et al*. using molecular dynamics simulations [31], random coils connecting the α-helices in HSA are strongly affected by the complexation with nanotubes, hence altering the protein tertiary structure. On the other hand, α-helices secondary structure is only slightly affected. Monte Carlo (MC) simulations confirmed the HSA distorted state when adsorbed to silver NPs. In this case, the Tryptophan residue, involved in intrinsic fluorescence, is quenched and situated at the protein boundary rather than the NP surface [32]. On flat graphite surfaces, a two-stage albumin complexation process was simulated [33]. In the initial stage, the protein was adsorbed with some loss of secondary structure, followed in the second step by protein reorientation and unfolding. Thus, the number of AAs in contact with the surface was maximized. At the microscopic scale, proteins are not necessarily rejected when arriving next to an occupied area on a flat surface, as shown by Monte Carlo simulations [34]. Instead, proteins can be tracked laterally within a certain distance due to the influence of pre-adsorbed proteins.

The development of theories can also contribute to the general knowledge of the protein field. For example, BSA interactions in the presence of salt may be modelled with a hard-core Yukawa potential, and the charge regulation mechanism involved in the complexation with charged NPs described by means of the Kirkwood–Shumaker theory [35,36].

Conformational and corona formation properties of simplified protein-like chains have already been investigated using Monte Carlo simulations [37,38]. The pH variation was systematically investigated. In this context, flexible polyampholytes enhanced the presence of dense conformations with optimized ion pairing. Moreover, the charge distribution on the chain significantly influenced its acid/base properties. The presence of charged NPs introduced extra electrostatic interactions, hence modifying the subtle interplay between attractive and repulsive interactions. Thus, specific conformations, such as electrostatic rosettes, were observed. In these previous studies, the sequence of blocks was alternated with various lengths, no counterions and no hydrophobic interactions were taken into account. Recently [39], the model was extended to investigate the effect of chain hydrophobicity and charge distribution in the interaction processes with NPs. Intermediate and hydrophilic backbones were found extended at extreme pH and folded at physiological pH. Moreover, they formed complexes with negatively-charged NPs at low pH and close to the chain isoelectric point, resulting from charge inhomogeneity. On the other hand, hydrophobic protein-like chains were not affected by the pH variation or NP presence. As a result, they remained folded and desorbed in all situations.

In this paper, we model a polypeptide (PP) chain and study corona formation with neutral and charged NPs. The BSA protein is used here as a model to describe the PP primary structure. The impact of salt valency and solution pH are systematically investigated, taking into account acid/base and hydrophobic properties of each AA. Research concerning serum albumins is very rich, mainly in the experimental domain. Computer simulations are also performed, usually to study specific aspects where pH variation is not involved. We propose here an original model to systematically follow the evolution of PP conformational and acid/base properties in presence of NPs, counterions, and salt. Here we explore in detail the individual charging behavior of each AA and their influence on complex formation with NPs, leading to denaturation.

## 2. Model

MC simulations were performed at a fixed temperature of 298 K in the grand canonical ensemble, according to the Metropolis algorithm [40]. An off-lattice three-dimensional coarse grained model was used to describe the system and the objects evolve in a cubic and periodic box (minimum image convention) with size of 2000 Å per side. The solvent was treated implicitly as a dielectric medium with relative dielectric permittivity constant $\varepsilon_{r}$ = 78.54 taken as that of water.

The system studied here was formed by one PP chain and one fixed NP surrounded by their counterions, as well as explicit salt particles when the effect of ionic strength *I* = 1 × 10^−4^ M is considered. All objects were described by impenetrable hard spheres to take into account the excluded volume effect. Counterions of PP and NP have a 2 Å radius with the charge located at their center (+1 or −1), and salt cations a 2.5 Å radius with charges of +1, +2, or +3. The system electro-neutrality is preserved by the addition of monovalent salt anions. The NP radius was set to 100 Å with a fixed and centered charge comprised between −471 and +471. The homogeneous surface charge density was then within the range σ = [−60, +60] mC/m^2^, consistent with systems composed of charged metal oxide NPs at physiological pH. The PP chain is represented as a succession of 583 freely jointed monomers of 2 Å radius. Each monomer corresponds to one AA, and they are distributed according to the BSA X-ray structure (Protein Data Bank, 3V03 [41]). Individual $pK_{a}^{}$ values of the AAs (amine and carboxylic acid groups, and also side-chain groups if present) were taken from the literature [42]. The charge of each AA, which is the total charge of the amine, carboxylic acid, and side-chain groups, is then pH dependent and varies from −1 to +1, −1 to +2, or −2 to +1, depending on the number of titrating sites and on the nature of the side-chain. Moreover, AAs such as Alanine (Ala), Methionine (Met), Leucine (Leu), Valine (Val), Isoleucine (Ile), and Phenylalanine (Phe) were considered hydrophobic [43]. Cysteine (Cys), which is a special case since the side-chain can act as a weak acid and form hydrogen bonds, was not included here as hydrophobic.

All pairs of charged objects *i* and *j* interact within the simulation box via a full Coulomb electrostatic and excluded volume potential, defined as

(1) $$U_{\text{ij}}^{\text{el}}\left(r_{\text{ij}}\right)=\{\begin{array}{cc} \infty , & r_{\text{ij}}<R_{i}+R_{j}\\ \frac{z_{i}z_{j}e^{2}}{4\pi \varepsilon_{0}\varepsilon_{r}r_{\text{ij}}}, & r_{\text{ij}}\geq R_{i}+R_{j} \end{array}$$

where e is the elementary charge (1.60 × 10^−19^ C), $\varepsilon_{0}$ the permittivity of the free space (8.85 × 10^−12^ CV^−1^m^−1^), $z_{\text{i},\text{j}}$ the charges carried by the AAs, NP, counterions, and salt particles, $r_{\text{ij}}$ the distance between them (center to center), and $R_{\text{i},\text{j}}$ their radii. $U_{\text{ij}}^{\text{el}}$ is then positive or negative when repulsive or attractive interactions occur. Hydrophobic interactions between Ala, Met, Leu, Val, Ile, and Phe are modelled through a 12-6 Lennard–Jones potential

(2) $$U_{\text{ij}}^{\text{vdW}}\left(r_{\text{ij}}\right)=\varepsilon_{\text{vdW}}\left[{\left(\frac{R_{i}+R_{j}}{r_{\text{ij}}}\right)}^{12}-2{\left(\frac{R_{i}+R_{j}}{r_{\text{ij}}}\right)}^{6}\right]$$

where $\varepsilon_{\text{vdW}}$ (in $k_{B}T$ units) is the minimum depth of potential located at distance $R_{i}+R_{j}$. $\varepsilon_{\text{vdW}}$ is set here to 3.5 $k_{B}T$ so as to observe folded conformations of isolated PP chains around physiological pH and extended (denatured) at extreme pH. Higher values of $\varepsilon_{\text{vdW}}$ do not lead to significant conformational changes with pH, which is not consistent with BSA experimental data [7,8]. Total energy $E_{\text{tot}}$ of the system is given by the sum of the whole pairwise potentials $U_{\text{ij}}^{\text{el}}$ and $U_{\text{ij}}^{\text{vdW}}$, taking into account periodic minimum image convention.

Conformations of low energy are under consideration in the Monte Carlo method. To reach these states, counterions, salt particles, and PP chains move through the box by translational movements. In addition, specific movements are applied to the chain, such as kink-jump, end-bond, reptation, and partially-clothed pivot [44,45,46]. Each movement is accepted or rejected according to the Metropolis algorithm, wherein $\Delta E_{\text{tot}}=E_{\text{tot}}^{\text{final}}-E_{\text{tot}}^{\text{initial}}$ plays a key role [40]. In the system presented here, the charge of each AA depends on solution pH. Thus, the state charge of N/4 random AAs is modified every 10,000 MC steps, according to one of the following schemes:

(3) $$\begin{array}{cccccccccc} \left(i\right) & & & -1 & ⇌ & 0 & ⇌ & +1 & & \\ \left(ii\right) & -2 & ⇌ & -1 & ⇌ & 0 & ⇌ & +1 & & \\ \left(iii\right) & & & -1 & ⇌ & 0 & ⇌ & +1 & ⇌ & +2 \end{array}$$

Case (i) is selected if the AA has only two titrating sites (amine and carboxylic acid groups), and cases (ii) or (iii) if the side-chain group is also pH dependent. The change of charge is random (right to left, or left to right), but only one titrating site is modified in the same time with the corresponding $pK_{a}^{}$ value. To keep the system electrostatically neutral, an oppositely-charged counterion is randomly inserted or removed if an additional charge appears or disappears on the PP backbone.

The acceptance of each AA protonation/deprotonation step is related to the MC Metropolis selection criterion [47,48]

(4) $$\Delta E=\Delta E_{\text{tot}}+a\cdot \chi \cdot k_{B}T\cdot \ln10\cdot \left(\text{pH}-pK_{a}\right)$$

where $k_{B}$ is the Boltzmann constant (1.38 × 10^−23^ JK^−1^) and T the temperature (298 K). The second term represents the change of free energy of the intrinsic association reaction of an AA. χ is equal to +1 or −1 if the titrating site of interest has acidic or basic behavior. The parameter a has a negative (−1) or positive (+1) value, depending on whether a charge (positive or negative) is inserted or removed on the AA, respectively. During the titration process, the system is coupled to proton and alkali baths (e.g., HCl and NaOH) in order to regulate the pH (input parameter) and provide explicit counterions (positive and negative). Simulations are carried out in grand canonical ensemble, the chemical potential (through $\text{pH}-pK_{a}$ values), box volume, and temperature remain fixed.

For a given pH value, an equilibration period (conformation relaxation) of 1 × 10^6^ MC steps is achieved, followed by a production period of 1 × 10^6^ steps. During this last part, macroscopic properties, such as radius of gyration, charge of the various species, radial distribution function (RDF), or OH^−^ equivalent to represent titration curves, are recorded to calculate ensemble averages. In addition, the layer of AA adsorption $Ads_{L}$ around the NP surface is defined as

(5) $$R_{\text{NP}}+R_{\text{AA}}\leq Ads_{L}\leq R_{\text{NP}}+3\times R_{\text{AA}}$$

with $R_{\text{NP}}$ and $R_{\text{AA}}$ the radii of the NP and AAs. The PP chain is considered adsorbed if at least one AA center is situated within the $Ads_{L}$ layer for more than 50% of the MC steps during the production period.

## 3. Results and discussion

### 3.1. Role of pH and NP Surface Charge Density in the Formation of Complexes

**Figure 1:**
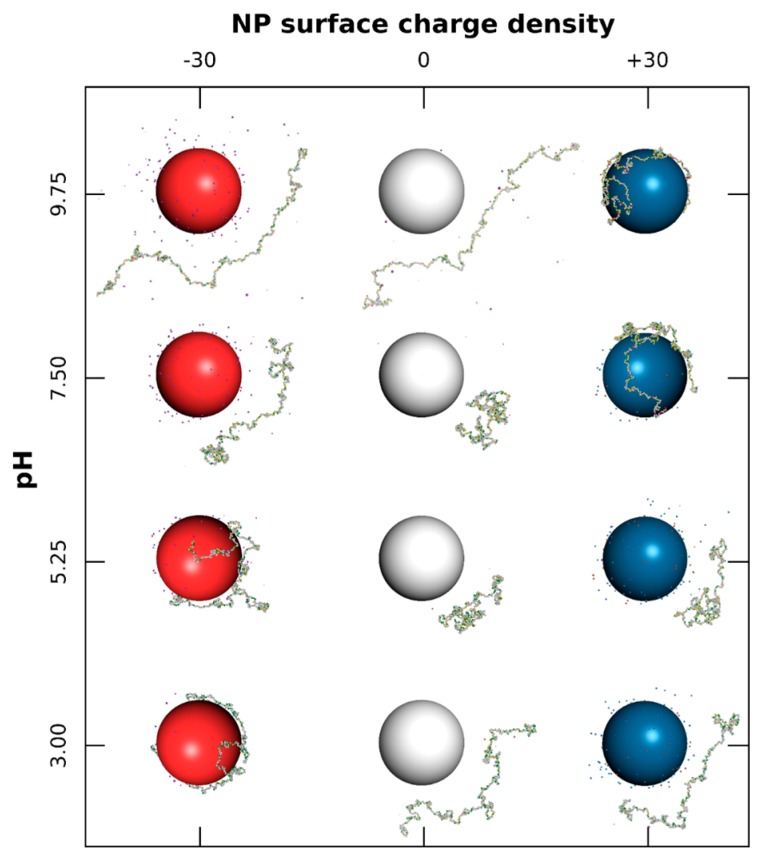
Monte Carlo simulations of polypeptide (PP) chain and nanoparticle (NP) surrounded by monovalent counterions. Negative, neutral, and positive NPs (100 Å radius) are represented by red, white, and blue spheres, respectively. AAs have green (positive), grey (neutral), and yellow (negative) colors. Cases with NP surface charge densities equal to −30, 0, and +30 mC/m^2^, and for pH variation from 3.00 to 9.75 are considered.

The interactions between one PP chain and one NP with surface charge densities σ ranging from −60 to +60 mC/m^2^, surrounded by positively and negatively charged monovalent counterions, are first investigated systematically under a range of pH (3.00 to 9.75). Equilibrated conformations are presented in Figure 1 for three σ and four pH values.

Globally, the PP chain is positively charged at low pH due to the protonation of basic (positively charged) and acidic (neutral) functional groups. Similarly, basic and acidic functional groups remain neutral and negatively charged at high pH, leading to a negative PP charge. At intermediate pH, all groups are charged, resulting in a globally neutral backbone. It has to be noted that the electrostatics (long-range interactions) are the main driving force inducing complex formation between PP and NP. Additionally, short-range interactions, such as hydrophobic, also play a key role in conformational variations of the chain. Therefore, equilibrated conformations of low energy are the result of subtle competitive interactions between PP with NP (electrostatic), PP and NP with their counterions (electrostatic) and AAs with AAs (electrostatic and hydrophobic).

As shown in Figure 1, the case with neutral NPs (middle column) indicates a strong influence of pH on PP conformation, and consequently on its acid/base properties. Here we consider only intra-chain and counterion interactions. The PP shows extended conformations (denaturation) at extreme pH due to repulsive electrostatic interactions between basic–basic and acidic–acidic functional groups at low and high pH, respectively. It has to be noted that hydrophobic interactions between AAs have only a limited effect on final conformations. At intermediate pH, effects of attractive electrostatic interactions (charged acidic and basic groups simultaneously present) as well as short-range hydrophobic interactions, result in locally folded conformations. This corresponds to the range where BSA protein can be found in native conformation N [49,50,51].

The complex formation with a negatively charged NP (Figure 1, first column) is observed at pH values of 5.25 and below. Indeed, the chain is positive and strong attractive electrostatic interactions occur with the NP surface. Meanwhile, the structure of PP is denatured, and the chain is wrapped around the NP, forming a corona structure. It has to be noted that hydrophobic interactions are responsible for locally folded segments which remain desorbed when PP is globally weakly charged (here at pH 5.25). The same general behavior is observed in the presence of positively charged NPs (Figure 1, third column); *i.e.*, the formation of complex when the PP chain is negatively charged (pH 7.5 and above). Thus, the PP is found here to be adsorbed only at the surface of positively charged NPs at physiological pH 7.5. Within the whole pH range investigated here, and by comparison with the neutral case (middle column), the presence of charged NP modifies the conformational behavior of the chain, and consequently its acid/base properties. Indeed, the formation of complexes limits PP structural changes (first column, pH 3–5.25, and third column, pH 7.5–9.75), and repulsive electrostatic interactions between NP surface and PP chain lead to more extended conformations when PP is desorbed (first column, pH 7.5, and third column, pH 5.25).

#### 3.1.1. Titration Curves

**Figure 2:**
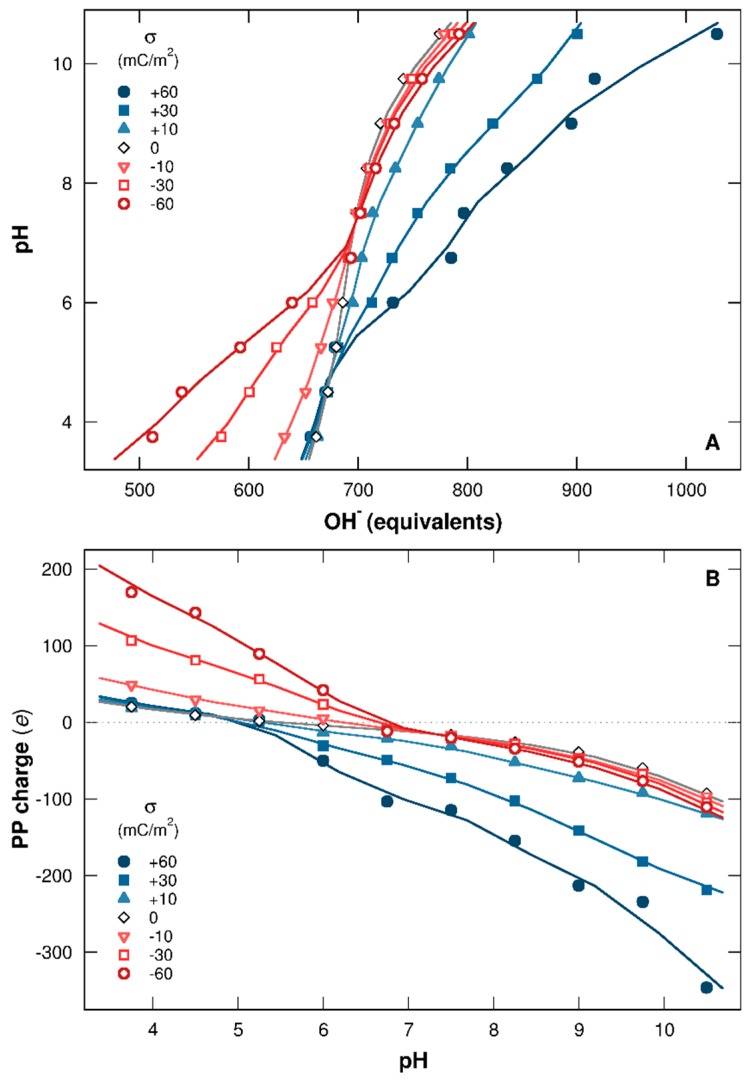
(**A**) PP titration curves (pH as a function of OH^−^ equivalents) and (**B**) total charges per chain (in elementary charges) calculated for NP surface charge densities ranging from −60 to +60 mC/m^2^. Red open symbols and blue closed symbols represent cases with negative and positive NPs. Neutral NP behavior is shown by black open symbols.

Figure 2A represents the titration curves of one PP chain in the presence of one NP having surface charge densities within the range [+60, −60] mC/m^2^. The pH is represented here as a function of base (in OH^−^ equivalents) necessary to neutralize the protons provided by the chain. Corresponding total charges per PP (in elementary charge unit) are calculated in Figure 2B as a function of pH.

It is clearly shown that the charged NP strongly influences the protonation/deprotonation behavior of AAs. When NP is neutral (Figure 2A, black open symbols), the curve remains symmetrical regarding to pH, and the deprotonation process is promoted by the increase of pH, resulting in an increase of OH^−^ equivalents to neutralize the protons. It has to be noted that the PP isoelectric point (pI) is 5.4 (Figure 2B), which is within the range 4.8–5.6 observed in other studies for BSA [6,52,53]. In general, pI values are dependent on the measurement techniques used and also on the ionic environment.

Charged NPs introduce extra electrostatic interactions in the system, leading to the loss of symmetry of titration curves (Figure 2A) as well as PP charge curves (Figure 2B) due to chain adsorption. In the presence of a negatively charged NP, the charging process of basic functional groups (positive charge) is promoted at low pH due to attractive electrostatic interactions with the NP. Consequently, the chain releases fewer protons to the bulk solution and less OH^−^ equivalents are necessary to neutralize them (Figure 2A, red open curves). Simultaneously, the total PP charge increases (Figure 2B, red open curves) and the system energy decreases. This behavior is only observed when complex formation is achieved (pH 7 and below). Thus, at high pH, the NP presence has no influence, since PP and NP are both negatively charged and not adsorbed. Similarly, the complex formation between PP and positively charged NP at high pH leads to the deprotonation process of AA acidic functional groups, hence favoring conformations of low energy. OH^−^ equivalents then increase (Figure 2A, blue closed curves), and the chain charge decreases (Figure 2B, blue closed curves). As the driving force here is the electrostatics, the interaction strengths are proportional to the amount of charges involved, leading to a more efficient PP charging process with stronger NP surface charge densities.

The pH range can be divided here in three domains in which PP acid/base properties and charging behaviors depend on the presence of (i) negatively charged NPs (pH 5.25 and below); (ii) positively charged NPs (pH 7 and above); and (iii) negatively and positively charged NPs (pH between 5.25 and 7). In this last pH range, isolated BSA proteins are known to be found in native conformation N [49,50,51].

#### 3.1.2. PP Chain Conformations

**Figure 3:**
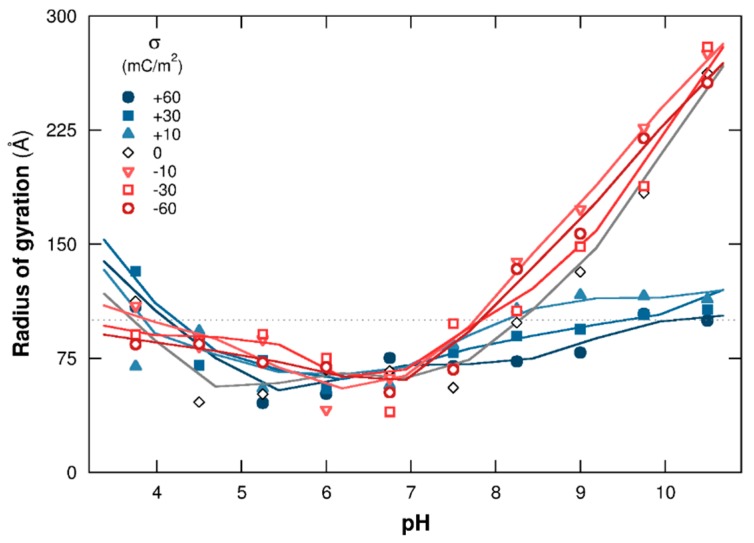
PP mean square radius of gyration as a function of pH. The chain is surrounded by a NP (σ = −60 to +60 mC/m^2^) and monovalent counterions. Cases with negative, neutral, and positive NPs are represented by red open, black open, and blue closed symbols. Dotted line is the NP radius (100 Å).

To capture the main features of PP structural changes regarding pH and NP surface charge density, the mean square radius of gyration is presented in Figure 3. Cases with negatively and positively charged NPs are shown with red open and blue closed symbols, respectively. Complex formation between the PP chain and NP strongly influences the evolution of chain structural changes. The radii of gyration increases here at extreme pH when no complex formation is observed; *i.e.*, pH 7 and above with negatively/neutral NPs and pH 5.25 and below with positively charged/neutral NPs. Indeed, PP is positively charged at low pH, resulting in strong repulsive electrostatic interactions with positively charged NPs, hence observing PP extended conformations. The same behavior is observed at high pH due to repulsive electrostatic interactions between negatively charged AAs and NPs. It has to be noted that the radius of gyration evolution, at low and high pH when no complex formation is observed, shows the same tendency compared to neutral NPs (black open symbols). The values are slightly larger with charged NPs due to additional repulsive electrostatic interactions between the PP chain and NP surface.

The formation of complexes at pH 7 and above with positively charged NPs and at pH 5.25 and below with negatively charged NPs limits PP structural changes. Indeed, positive and negative charges of the chain increase at low and high pH, respectively, and AAs are found fully adsorbed at the NP surface at extreme pH due to strong attractive electrostatic interactions with oppositely-charged NPs. As a result, the PP is wrapped around the NP and the radii of gyration evolve toward values corresponding to the NP radius (100 Å here).

At intermediate pH, between 5.25 and 7, the total PP charge is too low to form complexes. Indeed, the majority of PP functional groups are charged within this pH range, and positively charged AAs are counterbalanced by negatively charged ones, leading to a low or neutral total PP charge. In addition, the chain adopts folded conformations with small radii of gyration due to attractive electrostatic and hydrophobic interactions between AAs. No important PP structural changes are observed within this pH range, which is consistent with the native and folded conformations adopted by the BSA protein [49,50,51].

#### 3.1.3. Adsorption/Desorption Limits

**Figure 4:**
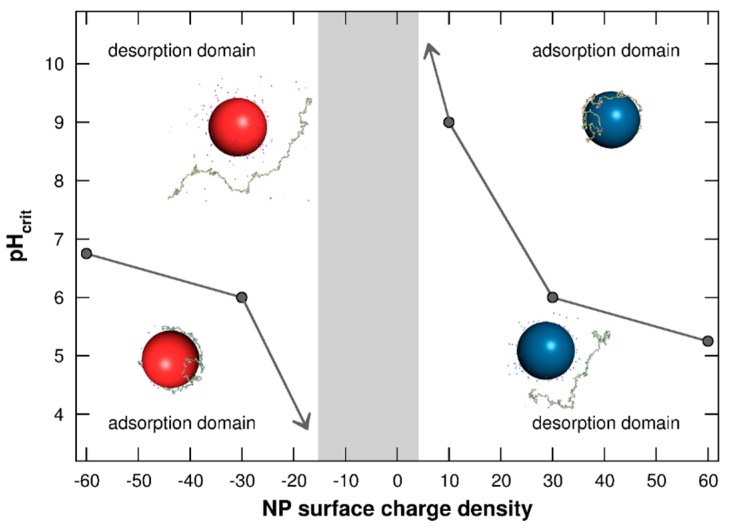
Adsorption/desorption limits pH_crit_ of PP chain situated in the NP adsorption layer as a function of NP surface charge density (σ = −60 to +60 mC/m^2^). Negative and positive NPs are represented by red and blue spheres. Monovalent counterions are present. The grey area shows the σ range in which no complex formation is observed.

The determination of adsorption/desorption domains between PP chain and NP surface is investigated in Figure 4 by representing the pH_crit_ as a function of NP surface charge densities σ = [−60, +60] mC/m^2^. The pH_crit_ is defined here as the pH value at which the chain is desorbed from the NP surface. As shown in Figure 3, PP conformations are strongly related to their capacity to form complexes with NPs. These adsorption/desorption diagrams are then of main importance to anticipate the conformational behavior and reactivity of PP chains in the presence of NPs.

It is shown in Figure 4 that pH_crit_ values are highly dependent on NP surface charge densities. At low pH, the PP adsorption process is promoted on negatively charged NPs, and complex formation between the negatively charged chain and positively charged NPs is favored at high pH. Considering negatively charged NPs, pH_crit_ decreases with the NP surface charge density tending towards 0. Indeed, weak attractive electrostatic interactions between both objects are observed with small σ values, hence promoting the AA desorption. Thus, the chain is only adsorbed when its charge is strongly positive—*i.e.*, at low pH. Similarly, pH_crit_ increases with σ decrease of positively charged NPs, hence favoring the formation of complexes only when PP acidic functional groups are fully deprotonated (high pH). The two adsorption domains are then reduced with smaller values of NP surface charge densities. Furthermore, a range of σ values is found between the two adsorption domains where complex formation is not observed (grey area). This feature is the result of very low attractive electrostatic interactions between the chain and NP due to weak NP surface charge densities or total PP charges.

### 3.2. Role of Salt Valency in the Formation of Complexes

We now investigate the complexation behavior of one PP chain and one charged NP carrying −471 or +471 elementary charges situated at its center (σ = −60 or +60 mC/m^2^). Explicit monovalent counterions (positive and negative), as well as monovalent, divalent, or trivalent salt particles, are present. Ionic strength has been fixed to *I* = 1 × 10^−4^ M, which is lower than in intra-cellular media [54]. The study of salt effect is important because the specific role and formation of biological complexes (e.g., chromatin compaction) can be modified with salt properties [55]. The influence of both salt valency and pH variation on PP adsorption is specifically studied here.

**Figure 5:**
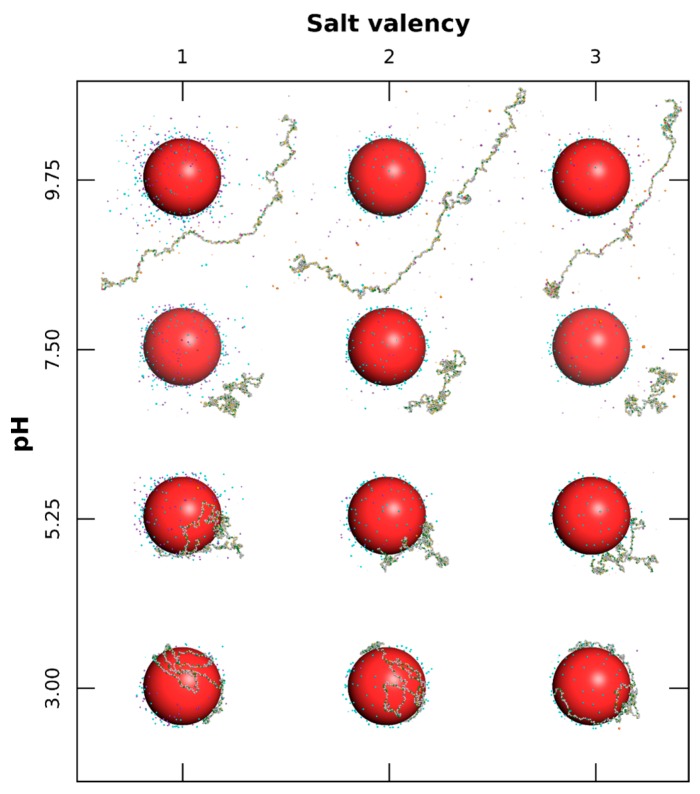
Equilibrated conformations of PP chain in the presence of a NP (100 Å radius) with surface charge density of −60 mC/m^2^. Each AA can be positive, neutral, or negative (green, grey, and yellow spheres). Monovalent counterions, as well as salt particles with fixed ionic strength of 1 × 10^−4^ M, are considered. The influence of pH variation (3.00 to 9.75) and salt valency (+1, +2, and +3) are specifically studied.

In Figure 5 equilibrated conformations are represented considering one negatively charged NP for various values of pH (3.00 to 9.75) and salt valencies (+1, +2, and +3). Globally, PP basic functional groups are protonated and positively charged at low pH in the presence of monovalent, divalent, or trivalent salt, hence promoting complex formation with the NP (pH 5.25 and below). No complex formation is observed at high pH due to repulsive long-range electrostatic interactions resulting from negatively-charged PP and NP. It is well known, through the DLVO theory [56], that the thickness of the electric double layer is influenced by salt properties and is compressed with an increase of salt valency due to stronger interactions with the NP surface. Such a screening effect of attractive electrostatic interactions between PP and NP is observed here by considering divalent or trivalent salt. Indeed, at pH 3, AAs are fully adsorbed as trains in the presence of monovalent salt and are partially desorbed as loops from the NP surface, considering trivalent cation salt. At pH 5.25, this behavior is more pronounced and AAs are mainly desorbed with trivalent salt. As a result, within the pH range of complex formation, attractive electrostatic interactions between NP and AAs are found in competition with interactions between NP and salt cations. It has to be noted that the complex structure is less dense at pH 5.25 due to the presence of more deprotonated acidic functional groups, leading to local electrostatic folded conformations.

Within the high pH range, the PP is negatively charged and no adsorption is observed. Salt cations are attracted here at the NP surface and by the chain so as to screen the repulsive interactions between AAs and to form locally small complexes. Folded PP segments are then observed at pH 9.75 in the presence of trivalent salt cations resulting from strong attractive electrostatic interactions.

#### 3.2.1. Titration Curves

**Figure 6:**
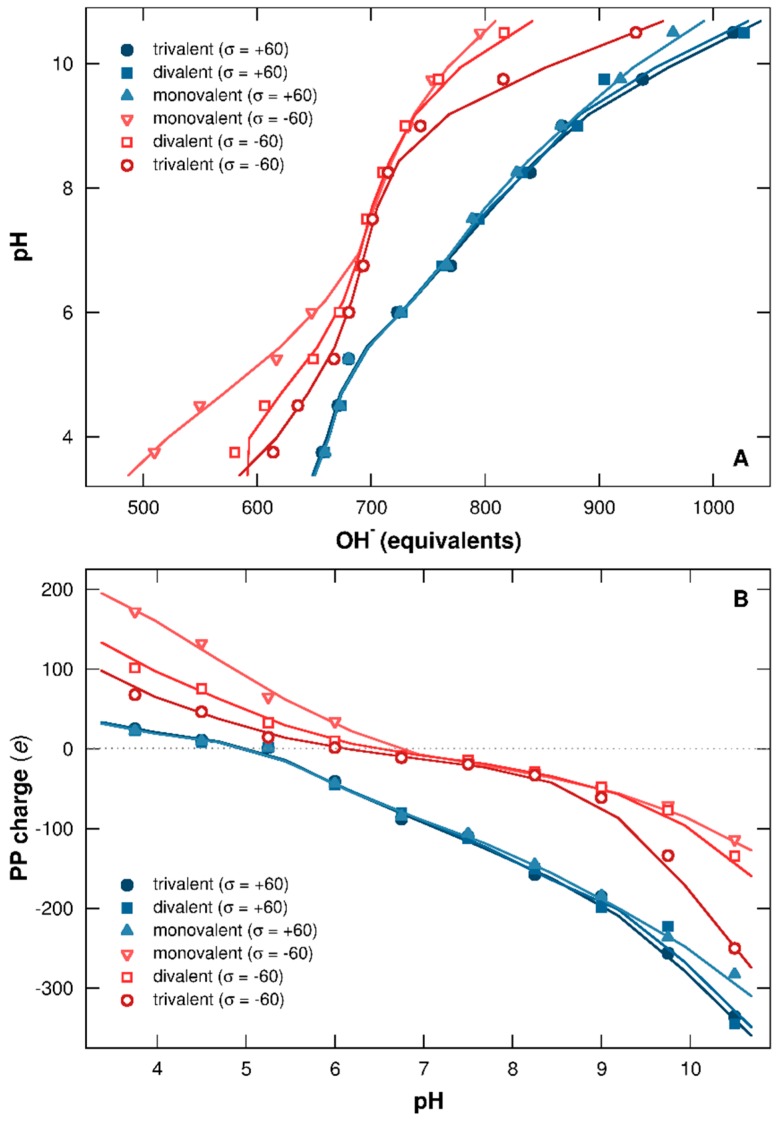
(**A**) PP titration curves (pH as a function of OH^−^ equivalents) and (**B**) total charges per chain (in elementary charges). NP surface charge densities are −60 (red open symbols) and +60 (blue closed symbols) mC/m^2^. Monovalent counterions and salt with variable valencies (+1, +2, or +3) are considered. Ionic strength is fixed to 1 × 10^−4^ M.

PP titration curves in the presence of one negatively or positively charged NP (σ = −60 or +60 mC/m^2^) are represented in Figure 6A, in which pH is given as a function of OH^−^ equivalents necessary to neutralize the protons released from the chain. PP charge is given as a function of pH in Figure 6B. Monovalent, divalent, or trivalent salts are considered to get an insight into the effect of salt valency.

It is found here that both salt valency and the sign of the NP surface charge are influencing the acid/base behavior of the chain. In Figure 6A, little variation is observed in the presence of positively charged NP (blue closed symbols). Salt has different effects at low and high pH. Indeed, salt cations are released into the bulk at low pH due to repulsive electrostatic interactions with NP and PP (both positively charged). In this case, the NP surface is only screened by monovalent salt anions and counterions, leading to overlapped titration and PP charge curves with the variation of salt valency (Figure 6A,B). At high pH, PP charge becomes negative (Figure 6B), promoting attractive electrostatic interactions with both NP and salt cations. Then the chain screening increases with salt valency, hence favoring the PP charging process. It has to be noted that PP is adsorbed at the positive NP surface at high pH, and only a limited number of salt cations are attracted to the chain due to repulsive electrostatic interactions between these cations and the NP, resulting in a small influence on PP acid/base properties.

The variation of salt valency in the presence of negatively charged NPs here shows a stronger impact on the PP protonation/deprotonation process (Figure 6A, red open curves). In this case, salt cations screen the NP surface, and their efficiency increases with their valencies. Within the range of complex formation, at low pH, attractive electrostatic interactions between NP with PP, and with salt cations, are in direct competition. Moreover, repulsive electrostatic interactions between salt cations and AAs are important here since all are attracted at the NP surface. Thus, with the increase of salt valency, the charging process of PP basic functional groups is less efficient, resulting in an increase of OH^−^ equivalents necessary to neutralize the PP protons remaining in solution. Therefore, the chain charge decreases (Figure 6B). At high pH, the PP is desorbed due to repulsive electrostatic interactions with the NP, but salt cations remain attracted around the chain and NP. In this case, the salt influences the deprotonation process of PP acidic functional groups, hence releasing more protons in solution with higher salt valencies (Figure 6A). During this process, PP charge increases, as shown in Figure 6B. Thus, acid/base properties of the chain in solution are strongly impacted by salt valency as well as the sign of NP surface charge.

#### 3.2.2. PP Chain Conformations

**Figure 7:**
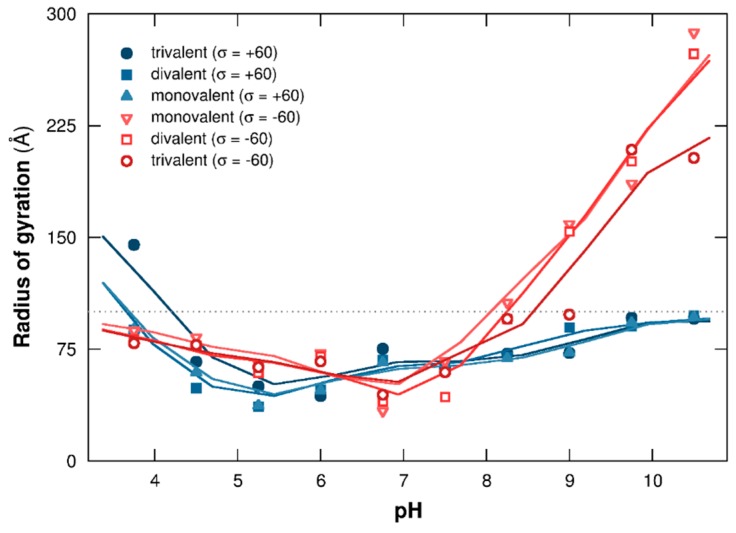
PP mean square radius of gyration as a function of pH. NPs with surface charge densities σ of −60 (red open symbols) and +60 (blue closed symbols) mC/m^2^, monovalent counterions and salt particles (valencies +1, +2, or +3) are considered. Ionic strength is fixed to 1 × 10^−4^ M, and the dotted line represents the NP radius (100 Å).

Evolution of PP structural changes in the presence of monovalent, divalent, or trivalent salt is now investigated via the calculation of the mean radius of gyration (Figure 7) and by considering one negatively or positively charged NP (σ = −60 or +60 mC/m^2^).

A similar trend is observed compared to the salt-free case in Figure 3. Indeed, the evolution of PP structural changes in Figure 7 is strongly limited by the adsorption of AAs at the NP surface (pH 7 and above with positive NPs, in blue closed symbols, pH 5.25 and below with negative NPs, in red open symbols). Within these two ranges, the PP chain is wrapped around the NP due to strong attractive electrostatic interactions, and the salt cation screening effect of NP or PP surfaces, when negatively or positively charged NPs are considered, is low even for higher salt valencies. Thus, ionic strength considered here (*I* = 1 × 10^−4^ M) is too low to destabilize electrostatic complexes formed by PP and NP, or to induce significant variation of chain structure.

When no complex formation occurs, at low and high pH in the presence of positive and negative NPs, respectively, the PP chain is extended with higher radii of gyration due to repulsive electrostatic interactions between AAs (Figure 7). The presence of higher salt valencies modifies the interaction range with the chain, resulting in small conformational changes. Indeed, PP charge is negative at high pH due to the deprotonation of the acidic functional groups. Thus, salt cations of higher valencies induce a stronger screening effect of repulsive electrostatic interactions between AAs, hence resulting in locally folded segments (see Figure 5, third column, pH 9.75) with smaller values of radius of gyration. It has to be noted that only trivalent salt has a real impact on conformational changes. The same behavior is observed considering a positively charged NP at low pH (Figure 7). In this case, salt cations introduce additional repulsive electrostatic interactions, stronger at high valencies, with AAs and NP. As a result, the PP chain adopts more extended conformations, especially with trivalent salt.

#### 3.2.3. Stability of Complexes

**Figure 8:**
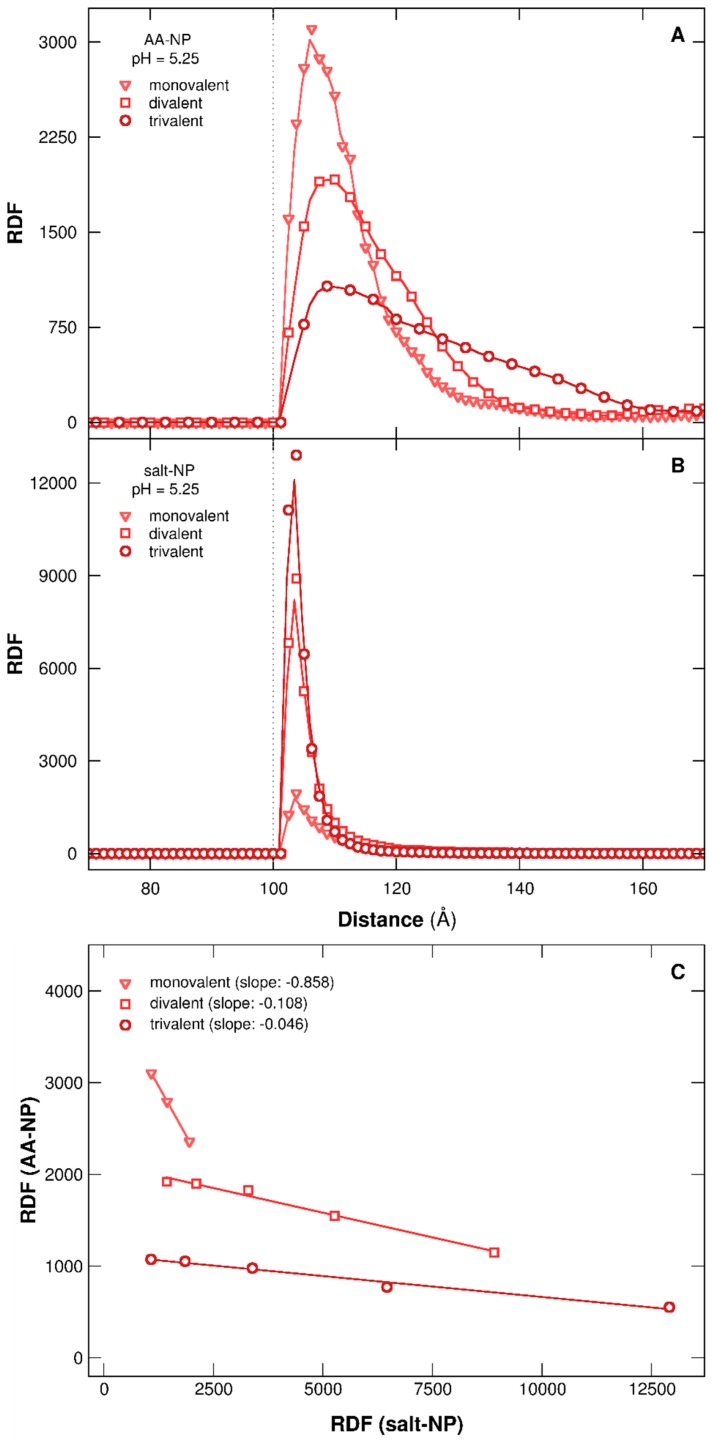
RDFs of (**A**) chain AAs and (**B**) salt cations around the NP surface (σ = −60 mC/m^2^) at pH 5.25. (**C**) represents the AA density as a function of salt cation density. Monovalent, divalent, or trivalent salts (ionic strength 1 × 10^−4^ M) are considered. Dotted lines represent the NP radius (100 Å).

The study of RDFs between the NP surface and the PP chain or salt cations (Figure 8) confirms the destabilizing effect of complexes by multivalent salt. RDFs are calculated here for one negatively charged NP at pH 5.25, in which complex formation is observed with monovalent, divalent, or trivalent salt. The evolution of AA density around NP, in Figure 8A, indicates a higher peak in the presence of monovalent salt due to stronger attractive interactions with NP. On the other hand, a small peak is obtained with trivalent salt, resulting from more efficient screening effects of NP surface, hence weakening the adsorption process of AAs. It has to be noted that RDF distribution is larger here since pH 5.25 represents the limit of AA adsorption/desorption considering trivalent salt. The AAs are then more labile around the surface. Salt distribution around the NP shows an opposite behavior (Figure 8B). Indeed, trivalent cations interact more strongly with the negatively charged NP compared to monovalent cations, resulting in a sharp peak. Furthermore, salt and AA densities at the NP surface are linearly correlated, as presented in Figure 8C. For the sake of clarity, only RDF values corresponding to the distance between the two peaks of adsorption, first salt–NP and second AA–NP, are presented. Indeed, we are interested here in corona stability situated in NP vicinity. We observe a strong variation of slopes in Figure 8C, considering the three types of salt. Thus, the higher slope, in absolute value, is found with monovalent salt. Consequently, the variation of salt density, in NP vicinity, induces an important change of AA density, hence confirming a denser corona structure with monovalent salt, and indirectly a stronger adsorption of AAs.

#### 3.2.4. Distribution of AAs at the NP Surface

**Figure 9:**
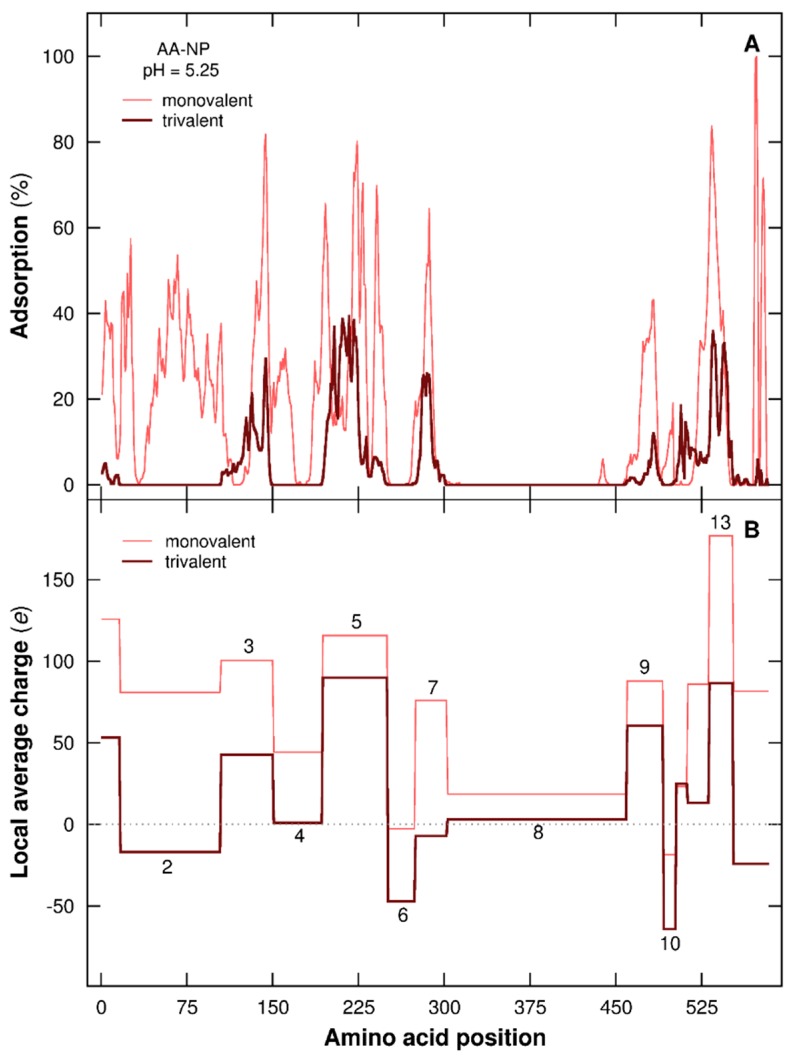
(**A**) Mean adsorption of each chain AA within the NP adsorption layer (in percentage), and (**B**) local charges per PP segment (in elementary charges). σ is −60 mC/m^2^ and pH 5.25. Monovalent and trivalent salt are considered (ionic strength 1 × 10^−4^ M). Domains 1–14 in (**B**) based on the adsorbed/desorbed PP domains in (**A**) considering monovalent salt. The same intervals are taken into account considering trivalent salt cations for comparison.

The PP primary structure plays a key role here in the adsorption/desorption processes of local chain segments. In Figure 9 the mean adsorption percentages of each AA within the adsorption layer of a negatively charged NP (σ = −60 mC/m^2^) and the corresponding local charges per PP segment at pH 5.25 are represented. These latter are based on adsorbed/desorbed chain domains in the presence of monovalent salt (Figure 9A), and the same intervals are taken into account considering trivalent salt for comparison. It has to be noted that the PP chain is adsorbed at pH 5.25 and exhibits more open conformations compared to desorbed chains (see Figure 7). Since the complex formation is here of electrostatic origin, the sequence of AAs and their acid/base properties directly influence the PP local charge (as shown in Figure 9B) and the ability to interact with the NP surface. When local segments are negatively charged or nearly neutral (domains 2, 4, 6, 8, and 10 with trivalent salt, domain 6 with monovalent salt), no significant adsorption is observed (Figure 9A). On the other hand, attractive interactions between AAs and NP occur for positively charged segments (domains 3, 5, 9, and 13 with trivalent salt, domains 2, 3, 4, 5, 7, 9, and 13 with monovalent salt). Comparing the properties of AAs, the most acidic ones are Aspartic Acid (Asp) and Glutamic Acid (Glu), and the most basic ones are Arginine, Lysine, and Histidine [42]. The percentage of these two AA groups present in each PP segment also influences the interactions with the NP. If we compare to the case involving monovalent salt, trivalent salt cations have more effects on the charge variation of PP segments carrying more Asp and Glu and with a higher positive local charge. Thus, the segments become less positive, or even negatively charged, hence modifying their adsorption at the NP surface. This behavior is illustrated in Figure 9A—e.g., domains 2 and 13, which have similar and strong variations between both salt cases. These two domains are built with 19.32% and 14.29% of acidic AAs (Asp and Glu). Even if the percentage of Asp and Glu is smaller in domain 13, charge variation is similar to domain 2 due to a more efficient charging process of both AAs resulting from a more positive local charge. In addition to these charging behaviors, the local surrounding environment is found to influence AA adsorption/desorption. Considering domains 7 (trivalent) and 10 (monovalent), local charges are weakly negative, which should promote desorption. Nonetheless, in Figure 9A, we observe an interaction with the negatively charged NP, which is the consequence of nearby adsorbed domains such as 5, 9, and 13. Moreover, a range of negatively and positively charged AAs are present at the same time, considering domains 7 and 10 with trivalent and monovalent salt, respectively, which facilitate their electrostatic matching. Domain 8 (monovalent) is here positively charged, and no adsorption is observed. Indeed, pairing between AAs due to electrostatic and hydrophobic attractive interactions is strong and stable, hence promoting a desorbed state (see Figure 5, pH 5.25).

#### 3.2.5. Adsorption/Desorption Limits

**Figure 10:**
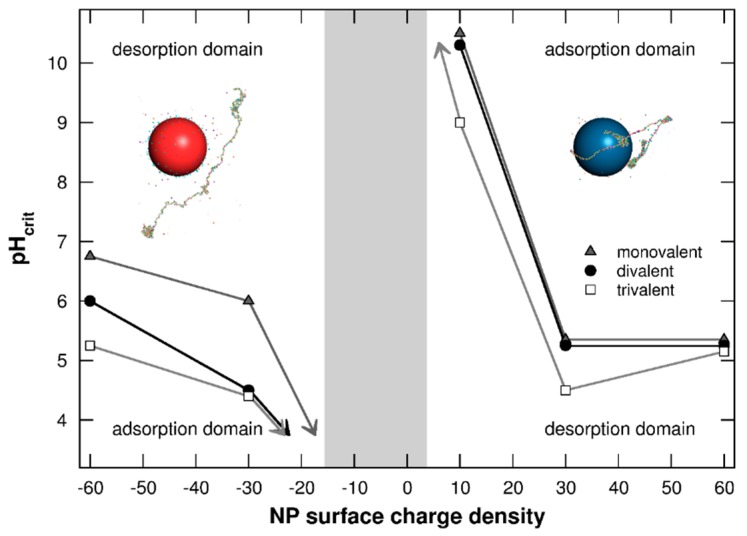
Adsorption/desorption limits pH_crit_ of PP chain situated in the NP adsorption layer as a function of NP surface charge density (σ = −60 to +60 mC/m^2^) in the presence of monovalent counterions. Red and blue spheres represent negative and positive NPs. Cases with monovalent, divalent, or trivalent salt cations are considered (ionic strength 1 × 10^−4^ M). Monovalent salt limit corresponds to the case without salt when NPs are negatively charged.

Adsorption/desorption limits (pH_crit_) of the PP chain at the NP surface are finally presented in Figure 10 in the presence of monovalent, divalent, or trivalent salt, and for various NP surface charge densities σ = [−60, +60] mC/m^2^. Similar behavior is observed with the three types of salt—*i.e.*, a decrease of adsorption domains for both negatively and positively charged NPs with the decrease of NP surface charge densities. Within the grey area situated between the two adsorption domains, PP attractive electrostatic interactions with NP are too weak to achieve the formation of complexes. Salt presence in the system modifies PP–NP interactions due to competitive effects with NP and chain surfaces, which are more efficient for high salt valencies. Considering negatively charged NPs, salt cations are situated at the NP surface and trivalent cations improve repulsive interactions with PP. Thus, the attraction of AAs within the NP adsorption layer is less effective, resulting in a decrease of the adsorption domain, as shown in Figure 10. The case with positively charged NPs shows a different behavior. Indeed, trivalent cations are situated around the chain, leading to a decrease of repulsive electrostatic interactions between AAs, and consequently to a more efficient deprotonation process of acidic functional groups. The PP attraction is then improved, resulting in a larger adsorption domain.

## 4. Conclusions

Metropolis MC simulations were carried out to investigate the influence of pH and salt valency in the formation of complexes involving a PP chain with primary structure based on BSA protein, and a positively or negatively charged NP. The coarse-grained model presented here showed its ability to reveal the role of key physicochemical parameters, such as pH, NP surface charge density, or presence of salt, driving the electrostatic complex formation.

The conformational properties of isolated PP chain evolved from extended at extreme pH to folded when the PP charge was neutral or weak. Several competitive additional electrostatic interactions were observed between AAs and with external compounds such as NPs, counterions, and salt particles, leading to an intricate conformational behavior.

When the PP chain was positively charged at low pH, the complex formation with negative NPs was promoted. On the other hand, when the chain was negatively charged at high pH, PP adsorption was favored in the presence of positive NPs. These complex formations resulted in a limitation of chain structural changes and improvement of PP charging processes, leading to a symmetry loss of titration curves. When desorbed, the NP presence did not significantly modify the PP acid/base properties. Most importantly, at physiological pH, the PP chain was adsorbed at the surface of positively charged NPs but not to negatively charged ones. 

The presence of salt in solution introduced additional competitive electrostatic interactions. The effect of salt cation valency, even at the low ionic strength investigated here, significantly influenced PP conformational and complex formation processes, mainly in the presence of negatively charged NPs. In this case, salt cations were located at the NP surface, hence screening the attractive interactions between the chain and NP. In addition, the charging process of PP became less efficient due to the nearby salt cations. In presence of trivalent salt, these two effects resulted in a decrease of the complex stability, leading to partially desorbed chain segments. It was also observed that some specific AAs had effects on the chain segments which were desorbed. The analysis of PP primary structure showed a stronger impact of trivalent cations on the charge of segments when strongly positively charged and when acidic AAs (Asp and Glu) were more abundant. The nearby surrounding environment was found to bias, in some cases, the AA adsorption/desorption within the NP adsorption layer.

Considering positively charged NPs, the increase of salt valency only had a limited impact on complex stability. Indeed, salt cations decreased the repulsive interactions between AAs, hence slightly promoting their charging process and interactions with positively charged NPs.

Systematic studies, as presented here, are of main importance because pH and ionic strength can be disrupted under cellular stress. These condition changes have the ability to induce significant conformational (and functionality) modifications which are important to evaluate. The next step of our model development will be the description of secondary structure (based on BSA protein), parametrization, and comparison with experimental systems including BSA proteins and NPs.
